# Pomalidomide promotes chemosensitization of pancreatic cancer by inhibition of NF-κB

**DOI:** 10.18632/oncotarget.24577

**Published:** 2018-02-26

**Authors:** Yoshihiro Shirai, Nobuhiro Saito, Tadashi Uwagawa, Hiroaki Shiba, Takashi Horiuchi, Ryota Iwase, Koichiro Haruki, Toya Ohashi, Katsuhiko Yanaga

**Affiliations:** ^1^ Department of Surgery, The Jikei University School of Medicine, Tokyo, Japan; ^2^ Division of Gene Therapy, Research Center for Medical Sciences, The Jikei University School of Medicine, Tokyo, Japan; ^3^ Division of Clinical Oncology and Hematology, Department of Internal Medicine, The Jikei University School of Medicine, Tokyo, Japan

**Keywords:** pancreatic cancer, pomalidomide, NF-κB, gemcitabine, S1

## Abstract

**Introduction:**

Nuclear factor κB (NF-κB) plays an important role in cancer progression and causes therapeutic resistance to chemotherapy. Pomalidomide, a third-generation immunomodulating drug derived from thalidomide, has been approved for uncontrolled multiple myeloma. We hypothesized that pomalidomide may inhibit the anticancer agent-induced NF-κB activity and enhance chemosensitization of combination chemotherapy with gemcitabine and S1 (Gem/S1) in pancreatic cancer.

**Methods:**

*In vitro*, we assessed NF-κB activity, induction of caspase cascade, cell apoptosis and cell proliferation using human pancreatic cancer cell lines (MIA PaCa-2 and PANC-1). *In vivo*, we established an orthotopic xenograft mouse model for human pancreatic cancer by injection of PANC-1 cells. At 5 weeks after injection, the animals were randomly divided into four groups and treated with Gem (100 mg/kg) /S1 (10 mg/kg), with oral administration of pomalidomide (0.5 mg/kg), with combination of gemcitabine, S1, and pomalidomide or vehicle only.

**Results:**

Although chemotherapeutic agents induced NF-κB activation in pancreatic cancer cells, pomalidomide inhibited anticancer agent-induced NF-κB activation (*p* < 0.01). Of the four groups tested for the apoptosis-related caspase signals and apoptosis under both *in vitro* and *in vivo* conditions, Gem/S1/Pomalidomide group demonstrated the strongest activation of the caspase signals and proapoptotic effect. In Gem/S1/Pomalidomide group, cell proliferation and tumor growth were slower than those in other groups both *in vitro* and *in vivo* (*p* < 0.01). There were no obvious adverse effects except for thrombocytosis by using pomalidomide.

**Conclusions:**

Pomalidomide promotes chemosensitization of pancreatic cancer by inhibiting chemotherapeutic agents-induced NF-κB activation.

## INTRODUCTION

Pancreatic cancer is the fourth leading cause of death in developed countries [1]. At the time of diagnosis, one-half of the patients present with metastatic disease and approximately 30% with unresectable locally advanced disease [2]. In addition, because of the rapid tumor growth and the high metastatic potential, the overall 5-year relative survival rate was only 10% [3]. Recently, the combination chemotherapy of gemcitabine and S1 (Gem/S1) is one of the most effective regimens for the treatment of locally advanced and metastatic pancreatic cancer in Korea and Japan [4]. However, the objective response rate of Gem/S1 therapy is limited to 30%, and the median survival time is only 10.1 months [5].

Transcriptional factor, nuclear factor kappa B (NF-κB) plays an important role in the regulation of cell proliferation, inflammation and oncogenesis [6], and constitutive NF-κB activation leads to the aggressive behavior of pancreatic cancer [7, 8]. In addition, many anti-cancer agents including gemcitabine induce NF-κB activation, which aggravates chemoresistance in cancer cells [9]. Therefore, control of chemotherapy-induced NF-κB activation could potentially enhance chemosensitivity. NF-κB consists of heterodimetric complex made up of the p65 (RelA) and p50 proteins, and normally presents in the cytoplasm as an inactive form with an inhibitor of NF-κB (IκBα) [10]. NF-κB activators including anticancer agents trigger the pathway run-through of IκBα phosphorylation by IκB kinase (IKK) complex, and NF-κB release from IκBα. The released NF-κB translocates into the nucleus and initiates target gene transcription and expression, including anti-apoptotic proteins such as cleaved inhibitor of apoptosis 1 (cIAP1), cIAP2, X-linked inhibitor of apoptosis, and Survivin [11]. We previously reported that nafamostat mesilate, a synthetic serine protease inhibitor, inhibited gemcitabine-induced activation of NF-κB and increased chemosensitivity to gemcitabine and cell apoptosis in pancreatic cancer [12].

Pomalidomide is one of the third generation immunomodulating drugs (IMiDs), which is derived from thalidomide and has been approved in the United States for the treatment of relapsed or refractory multiple myeloma [13]. Anticancer effects of IMiDs in hematologic malignancies have been reported including suppression of angiogenesis, as well as immunomodulation and inhibition of cytokine production [14]. For multiple myeloma, pomalidomide induced G0/G1 cell cycle arrest by upregulating the expression of p21^waf1^ [15], anti-angiogenesis by multiple inhibition of endothelial cell function [16] and apoptosis through upregulation of caspase-8 by inhibiting the expression of IAP2 and tumor necrosis factor (TNF)-related ligand [17]. Furthermore, thalidomide, a predecessor of pomalidomide, exerts suppression of NF-κB activation and apoptosis induction via caspase cascade [18]. Although a few reports described pomalidomide as a NF-κB inhibitor in multiple myeloma, the role of pomalidomide in pancreatic cancer has not been investigated.

The purpose of this study is to demonstrate that pomalidomide enhances chemosensitization of pancreatic cancer by inhibition of Gem/S1-induced NF-κB activation *in vitro* and *in vivo*.

## RESULTS

### Pomalidomide improved chemosensitivity in human pancreatic cancer cells

**Figure 1:**
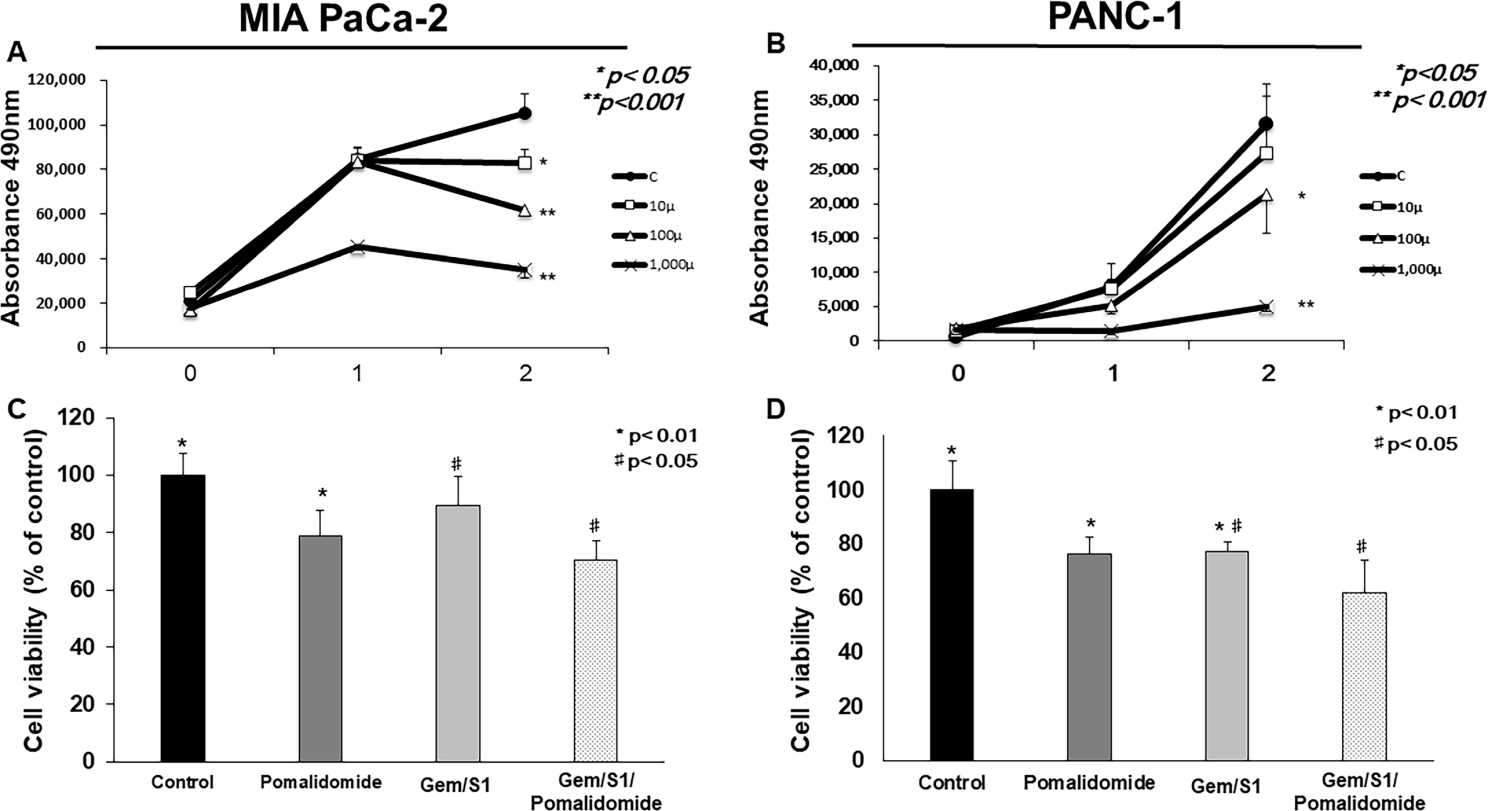
The cell viabilities of MIA PaCa-2 and PANC-1 cells were measured by the MTT assay (**A**, **B**) Pomalidomide monotherapy (100 μM and 1 mM) showed antiproliferative effects in 48 hours (*p* < 0.05 and *p* < 0.001, respectively). The cell viabilities in the Gem/S1/Pomalidomide were less than those in Gem/S1 (**C**) MIA PaCa-2: 70.5 ± 6.7 vs. 89.3 ± 10.2%, *p* < 0.05, (**D**) PANC-1: 62.2 ± 11.8 vs. 77.2 ± 10.7%, *p* < 0.01.

To investigate the effect of pomalidomide monotherapy on cell proliferation in pancreatic cancer, we examined cell viabilities by MTT assay. Although low dose of pomalidomide (10 μM) had anti-proliferative effects only in MIA PaCa-2 cells, the concentration (100 μM) used in myeloma experiment and high dose (1 mM) of pomalidomide demonstrated anti-proliferative effects in both pancreatic cancer cells (Figure 1A, 1B). Next, we examined cell viabilities after the treatment with Gem/S1 with or without pomalidomide to verify the additive effect of pomalidomide. In results, pomalidomide enhanced Gem/S1 therapy-induced anti-proliferative effects in both cell lines (*p* < 0.01 each) (Figure 1C, 1D). These indicated that high dose of pomalidomide showed the anti-proliferative effects and improved chemosensitivity of human pancreatic cancer cells. In addition, we calculated combination index (CI) between gemcitabine and pomalidomide (data not shown). In both cell line, CI was over 1, which suggested that pomalidomide has only additive effects.

### Enhancement of apoptosis signals and induction of apoptosis by pomalidomide

**Figure 2:**
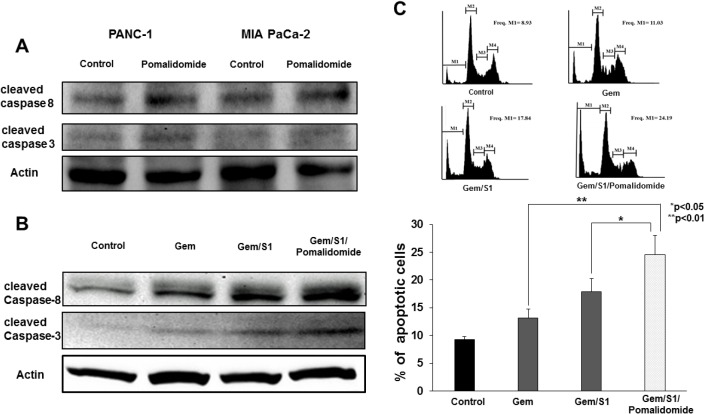
Western blot analysis demonstrated the expression levels of protein related to apoptosis signals at 24 hours (**A**) The expression levels of cleaved caspase-8 and -3 were comparable between those with or without pomalidomide. (**B**) Gem/S1/Pomalidomide increased the expression levels of cleaved caspase-8 and -3. (**C**) Cell cycle analysis showed apoptosis cells after each treatment for 48 hours. M1, M2, M3, and M4 signify sub-G1, G0/G1, S, and G2/M phases, respectively. Gem/S1/Pomalidomide had the largest apoptotic cell population, which was larger than Gem/S1 (24.6 ± 3.4 vs. 17.9 ± 2.4%, *p* < 0.05).

To clarify the mechanism of the improvement of chemosensitization to Gem/S1-induced anti-proliferative effect by pomalidomide, we next analyzed induction of apoptosis signals by Western blots and flow cytometry. Firstly, we examined the effect of pomalidomide monotherapy on the expression levels of cleave caspase-8 and -3 (Figure 2A). In results, pomalidomide monotherapy did not show clear effects of apoptosis induction. On the contrary, in combination therapy, pomalidomide enhanced the expression levels of cleaved caspase-8 and cleaved caspase-3 protein in Gem/S1/Pomalidomide group compared with that in Gem/S1 group (Figure 2B). Subsequently, we measured the cell population of sub-G1 cells using cell cycle analysis, which indicated apoptotic cells. As a result of induction of apoptosis signals, Gem/S1/Pomalidomide group had the largest sub-G1 cell population (Figure 2C). These indicated that pomalidomide monotherapy had little effects on cell apoptosis, whereas addition of pomalidomide to Gem/S1 therapy increased Gem/S1-induced expression levels of apoptosis signals and apoptotic cells.

### Pomalidomide inhibited anti-cancer agents-induced NF-κB activation

**Figure 3:**
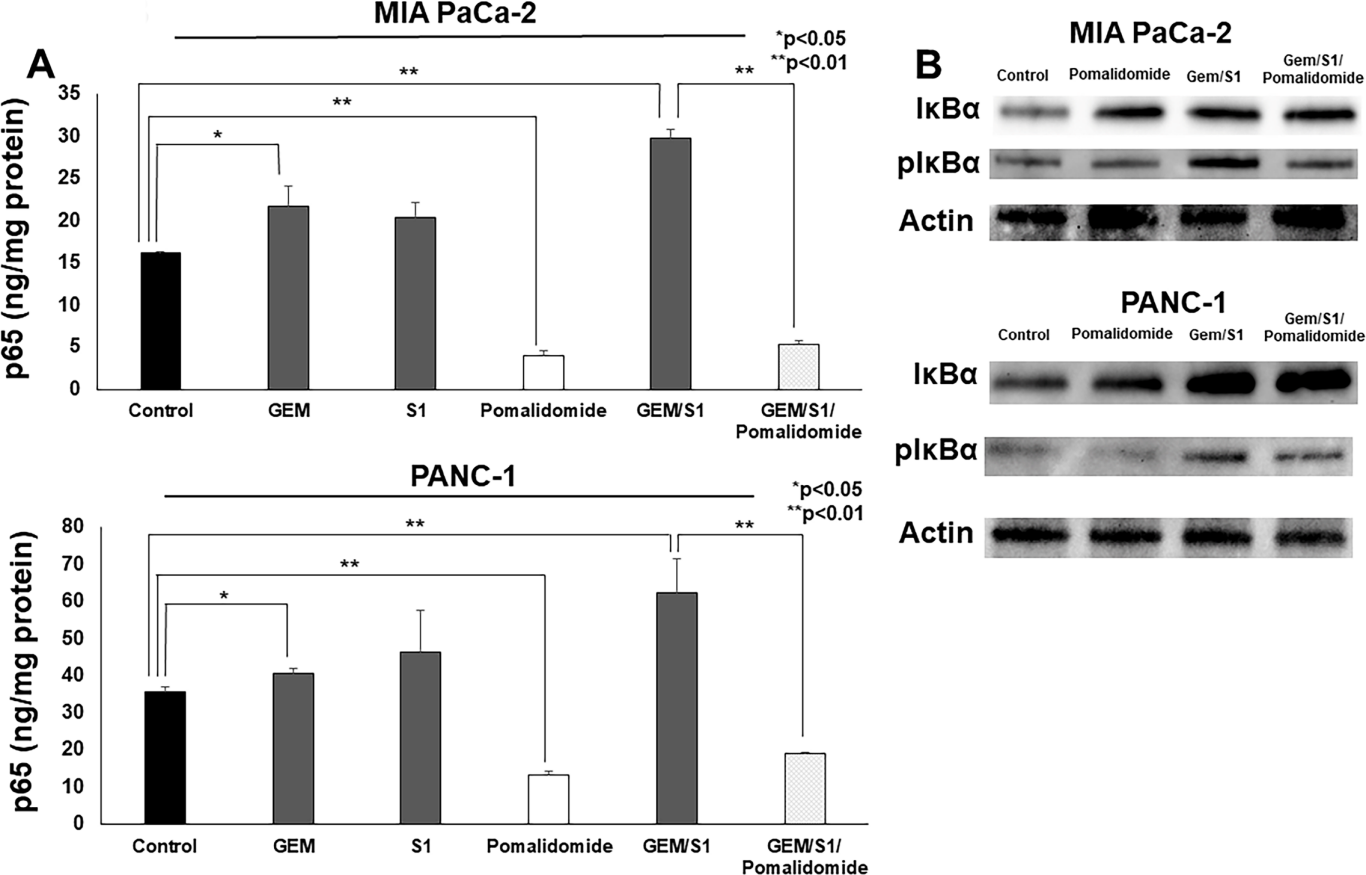
(**A**) The ELISA assay revealed that the NF-κB p65 concentration of MIA PaCa-2 and PANC-1 cells treated with Gem or Gem/S1 for 2 hours were higher than control (*p* < 0.05), whereas pomalidomide decreased the p65 concentration compared with control group at 2 hours (*p* < 0.01). p65 concentration in Gem/S1/Pomalidomide was less than that in Gem/S1 (*p* < 0.01). (**B**) Western blots showed that the expression levels of phosphorylated IκBα were decreased in pomalidomide administrated group.

To investigate more details of apoptosis induction, we examined the role of pomalidomide associated with NF-κB activation, which increases the expression of anti-apoptotic proteins. As the localization of nucleus in the pancreatic cancer cells reflects NF-κB activation, the concentration of p65, the subunit of NF-κB dimers, in the nuclear extracts were measured. *In vitro*, anti-cancer agents (Gem or Gem/S1) increased the activation of NF-κB at 2 hours after treatment in both cell lines (*p* < 0.05 and *p* < 0.01, respectively) (Figure 3A). On the contrary, pomalidomide strongly inhibited the translocation of NF-κB from the cytoplasm into the nucleus in pancreatic cancer cells (*p* < 0.01). Furthermore, pomalidomide inhibited anti-cancer agents-induced NF-κB activation (*p* < 0.01). To investigate more details of the inhibitory mechanism of NF-κB activation by pomalidomide, we evaluated the expression levels of selective upstream molecules such as IκBα base and phosphorylated IκBα (Figure 3B). Although the levels of IκBα base were comparable between Gem/S1 and Gem/S1/Pomalidomide, pomalidomide decreased the expression levels of phosphorylated IκBα. These results suggested that pomalidomide inhibits phosphorylation of IκBα and subsequently suppresses NF-κB activation. In results, pomalidomide enhanced Gem/S1 therapy-induced cell apoptosis and antiproliferative effects in pancreatic cancer cells.

### Pomalidomide enhanced anti-tumor effect of Gem/S1 in orthotopic xenograft mouse model

**Figure 4:**
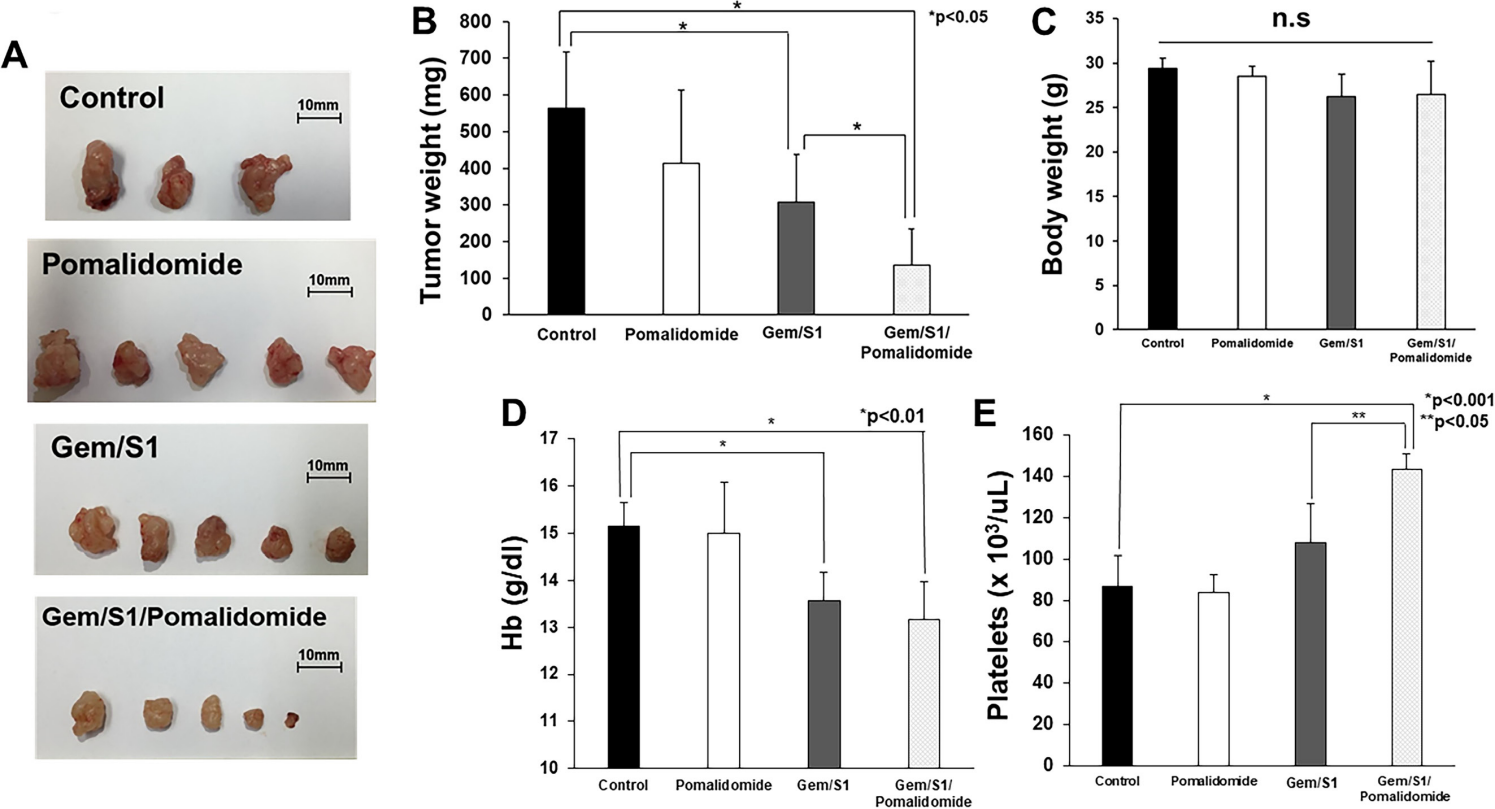
(**A**) Gem/S1/Pomalidomide had the smallest tumors after 6 weeks of treatment. (**B**) The excised tumor weight in Gem/S1/Pomalidomide was lower than Gem/S1 (137.2 ± 98.4 vs. 308.6 ± 128.7 mg, *p* < 0.05). (**C**) Body weight after 6 weeks of treatment was comparable in 4 groups. (**D**) Hemoglobin in Gem/S1 with and without pomalidomide were lower than that in control (Gem/S1 vs. Gem/S1/Pomalidomide vs. Control = 13.6 ± 0.5 vs. 13.2 ± 0.8 vs. 15.5 ± 0.5, *p* < 0.01). (**E**) The platelet counts in Gem/S1/Pomalidomide was higher than that in control (*p* < 0.001) or GEM/S1 (*p* < 0.05).

To assess the anti-tumor activity by Gem/S1 with pomalidomide *in vivo*, nude mice bearing xenograft were randomly divided into four groups (Control, Pomalidomide, Gem/S1, and Gem/S1/Pomalidomide). Single-treatment of pomalidomide showed only a mild anti-tumor effect in a mice model, while Gem/S1 therapy suppressed tumor growth compared with control (*p* < 0.05) (Figure 4A, 4B). Furthermore, pomalidomide enhanced anti-tumor effect of Gem/S1 (*p* < 0.05). After 6 weeks of these treatments, the tumor weight in Gem/S1/Pomalidomide group were reduced by more than 50% of that in Gem/S1 group. There were no significant differences in the body weight of the animals among the four groups (Figure 4C). We also assessed the effect of those treatments on hemoglobin (Hb) and platelets. Although Gem/S1 and Gem/S1/Pomalidomide groups demonstrated anemia (*p* < 0.01), Hb was comparable between Gem/S1 and Gem/S1/Pomalidomide groups (Figure 4D). The platelet counts in Gem/S1/Pomalidemide group was higher than that in control or Gem/S1 group (*p* < 0.05) (Figure 4E).

### Pomalidomide enhanced anticancer agents-induced tumor suppressor *in vivo*

**Figure 5:**
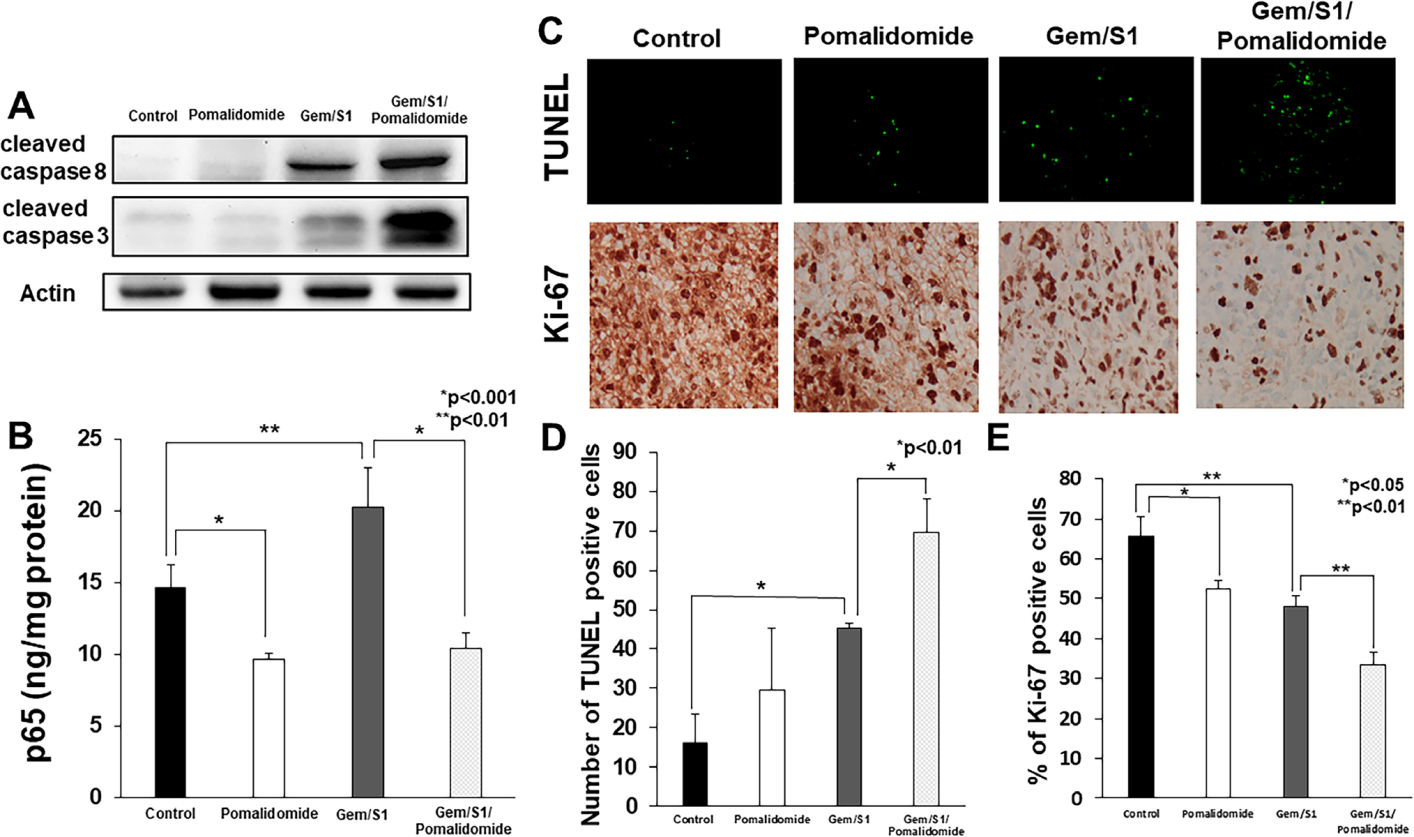
(**A**) Western blot analysis demonstrated the expression of apoptosis-related proteins in the excised tumor tissues. The expression levels of cleaved caspase-8 and -3 in Gem/S1/Pomalidomide were higher than those in the other groups. (**B**) In assessment of NF-κB p65 activation by ELISA, the concentration of p65 in the nuclear extract of excised tumor tissues in pomalidomide group was lower than control (*p* < 0.001), whereas the level of p65 was greater in Gem/S1 (*p* < 0.01). In Gem/S1/Pomalidomide, p65 concentration was lower than that in Gem/S1 (*p* < 0.001). (**C**) In TUNEL staining of the excised tumor tissues, (**D**) the number of TUNEL-positive cells in the Gem/S1/Pomalidomide was greater than that in Gem/S1 (69.7 ± 8.4 vs. 45.3 ± 1.2, *p* < 0.01). (**E**) The percentage of Ki-67-positive cells in Pomalidomide was lower than those in control (52.6 ± 2.1 vs. 65.6 ± 4.9%, *p* < 0.05), and those in Gem/S1/Pomalidiomide was lower than those in Gem/S1 (33.4 ± 3.3 vs. 48.1 ± 2.5%, *p* < 0.01), respectively.

The effects of pomalidomide on NF-κB activation and apoptosis in pancreatic tumor were assessed using resected specimens. To verify the *in vitro* findings regarding the roles of pomalidomide, *in vivo* expression levels of apoptosis-related proteins and NF-κB activation were assessed. Consistent with the *in vitro* data, these protein expression patterns in xenograft tumors treated with Gem/S1/Pomalidomide showed a pattern similar to those observed *in vitro* (Figure 5A).

The TUNEL assay of the removed tumor that stain apoptotic cell demonstrated striking similarity with our *in vitro* study result that addition of pomalidomide further potentiated apoptosis induced by Gem/S1 (Figure 5C). Indeed, when the number of TUNEL positive cells was counted, not only Gem/S1 demonstrated significantly more apoptotic cells, but addition of pomalidomide demonstrated significantly more apoptotic cells compared from Gem/S1 alone (Figure 5D). Similar to *in vitro* experiments, pomalidomide also suppressed the activation of NF-κB (Figure 5B). Immunohistochemical staining of Ki-67 showed that pomalidomide alone demonstrated significant suppression of cancer cell proliferation as well as Gem/S1 alone group (Figure 5E). Addition of pomalidomide to Gem/S1 demonstrated further significant suppression of cell proliferation, which implicate that Gem/S1/Pomalidomide combination therapy not only significantly increase apoptosis, but also suppress cancer cell proliferation.

## DISCUSSION

For the last two decades, gemcitabine has been the standard regimen for unresectable pancreatic cancer. Only recently, FOLFIRINOX (oxaliplatin, irinotecan, fluorouracil, and leucovorin) has been reported to be superior to gemcitabine monotherapy in 2011 [19]. The combination therapy of gemcitabine and nab-paclitaxel also had superiority over gemcitabine alone [20]. Also, the advantage of combination chemotherapy of gemcitabine and S1 over gemcitabine alone for advanced pancreatic cancer has been reported [5]. Improving these treatment with NF-κB inhibitors could be a new standard therapy for unresectable pancreatic cancer. There were several clinical reports of combination therapy targeting NF-κB for pancreatic cancer by using NF-κB inhibitors such as a curcumin [21], nafamostat mesilate [22, 23], bortezomib [24], thalidomide [25], and lenalidomide [26]. These clinical trials had acceptable outcomes. We previously reported that combination chemotherapy of gemcitabine and nafamostat mesilate, a NF-κB inhibitor, had favorable outcomes for unresectable pancreatic cancer in phase 1 and 2 study [22, 23]. However, nafamostat mesilate did require intra-arterial administration via a reservoir port, which necessitates infusion therapy at a hospital or outpatient clinic. On the other hands, as pomalidomide is orally administered, which could significantly reduce physical and financial burden to patients. Therefore, we selected pomalidomide as a novel NF-κB inhibitor for the combination chemotherapy. Although phase 1 clinical trial about combination therapy of pomalidomide and gemcitabine was performed [27], the correlation between NF-κB and pomalidomde was not clear.

Thalidomide, the first generation IMiDs, suppresses NF-κB activation via inhibition of IκBα phosphorylation [18]. In the present study, we found that pomalidomide which is a third generation IMiDs derived from thalidomide has similar effect on IκBα phosphorylation in pancreatic cancer cells. Furthermore, pomalidomide enhanced gemcitabine and S1-induced apoptotic signals and apoptosis. Consequently, pomalidomide demonstrated enhanced gemcitabine and S1-induced antitumor effects *in vivo*, suggesting that triple combination therapy of gemcitabine, S1 and pomalidomide had a synergistic anticancer effect on pancreatic cancer. To our knowledge, this is the first report of novel function of pomalidomide as an NF-κB inhibitor in non-hematologic malignancies.

Pomalidomide monotherapy demonstrated significant anti-proliferative effect *in vitro* and mild anti-tumor effect *in vivo*. However, pomalidomide monotherapy have no effects on induction of apoptosis. We hypothesized that other effects of pomalidomide which were reported previously such as anti-angiogenesis, immunomodulation, or cell cycle arrest may function in this study. Immunohistochemical staining showed that Ki-67 positive cells were decreased in pomalidomide monotherapy group compared with control (Figure 5C, 5E), which suggested that pomalidomide induced cell cycle arrest in pancreatic cancer. In these points, the effects of pomalidomide remain unclear in pancreatic cancer, so further studies are required.

Pomalidomide originally has been used for multiple myeloma with dexamethasone. The safety of pomalidomide has been validated in several clinical trials. Therefore, this combination therapy could easily be applicable to clinical practice. Several adverse effects have been reported in the combination with pomalidomide and dexamethasone for the patients with multiple myeloma. The most common grade 3 or 4 toxicities were primarily neutropenia, which occurred in 32–65% of the patients [28–30]. Other hematological grade adverse effect was anemia in 5–36% and thrombocytopenia in 3–31% [28–30]. Venous thromboembolic events were reported as an important non-hematological adverse effect. Therefore, all patients receiving pomalidomide therapy were required to administer thromboprophylaxis, which reduced the rate of venous thromboembolic events to 2–3% [28–30]. In the current study, both of combination therapy of Gem/S1 with or without pomalidomide showed anemia by 6 weeks of therapy, but there were no significant differences between Gem/S1 with pomalidomide and Gem/S1 group (Figure 4D). On the contrary, Gem/S1/Pomalidomide therapy demonstrated thrombocytosis, which suggested thrombotic tendency (Figure 4E). Therefore, the patients with pancreatic cancer may require thromboprophylaxis during the therapy.

In summary, the current study demonstrated that pomalidomide enhanced Gem/S1-induced cell apoptosis by inhibiting NF-κB activation in pancreatic cancer *in vitro* and *in vivo*. IMiDs could be a novel potential option for chemotherapy in pancreatic cancer.

## MATERIALS AND METHODS

### Cell culture

Human pancreatic cancer cell lines MIA PaCa-2 and PANC-1 were purchased from American Type Culture Collection (Rockville, MD, USA). Both cell lines were maintained in Dulbecco’s modified Eagle’s Medium (DMEM) containing 10% fetal bovine serum (Thermo Fisher Scientific, Rockville, MD, USA) and 1% penicillin/streptomycin (Thermo Fisher Scientific). The cells cultured at 37° C with 5% CO_2_. Cells were given fresh media before treatment.

### Reagents

Pomalidomide (Sigma-Aldrich Corp., Missouri, USA) was dissolved in DMSO (10 mg/ml) and stored at −80° C until use. S1 contains a combination of FT (5-fluoro-1-(tetrahydro-2-furfuryl) uracil, Tokyo Chemical Industry, Tokyo, Japan) and Gimeracil (5-chloro-2, 4-diharroxypridine, Tokyo Chemical Industry) at a molar ratio of 1:0.4 dissolved in 0.5% hydroxypropylmethylcellolose (HPMC; Shin-Etsu Chemical Co., Ltd., Tokyo, Japan). Gemcitabine was purchased from Eli Lilly Japan (Tokyo, Japan).

### Antibodies

Antibodies specific to cleaved caspase-8 and -3 were obtained from Cell Signaling Technology (Beverly, MA, USA). Anti-β-actin antibody was purchased from Sigma Chemical (St. Louis, MO, USA).

### *In vitro* experimental treatment groups

On the basis of previous study, MIAPaCa-2 and PANC-1 cells were treated with gemcitabine (10 nM) (Gem), both gemcitabine (10 nM) and S1 (0.25 μg/ml) (Gem/S1), gemcitabine (10 nM), S1 (0.25 μg/ml), and pomalidomide (100 μM) (Gem/S1/Pomalidomide), and vehicle-only (control) for the appropriate time in each analysis [31]. In Gem/S1/Pomalidomide group, pancreatic cancer cells were treated with pomalidomide for 2 hours before gemcitabine and S1 treatment.

### Animals and *in vivo* orthotopic experimental models

Five-week-old male nude mice (BALBc nu/nu) purchased from CLEA Japan Inc. (Tokyo, Japan) were housed under specific pathogen-free conditions in a biologic cabinet at the Laboratory Animal Facility of The Jikei University School of Medicine. We established an orthotopic pancreatic cancer xenograft model in mice by injection of 5.0 × 10^6^ PANC-1 cells suspended in 50 μL of phosphate buffer saline (PBS) into the tail of the pancreas. At 5 weeks after injection, the animals were treated with oral administration of pomalidomide (0.5 mg/kg) three times a week (Pomalidomide group), intravenous (i.v.) injection of Gemcitabine (100 mg/kg) once a week in condition with oral administration of S1 (10 mg/kg) three times a week (Gem/S1 group), or combination therapy of Gemcitabine, S1, and pomalidomide (Gem/S1/Pomalidomide group). For control group, the equal amount of distilled water was injected (i.v.) weekly and 5% of DMSO (p.o.) three times a week. To measure full blood counts as the toxicity of treatment by Celltac α MEK-6358 (NIHON KOHDEN, Tokyo, Japan), blood samples were taken at 6 weeks after treatment. At 6 weeks after treatment, the animals were humanely killed and the pancreatic tumor were excised. This research was approved by the Institutional Animal Care and Use Committee of The Jikei University School of Medicine (2015-002).

### Cell proliferation assay

Pancreatic cancer cells were seeded into 96-wells plates (5 × 10^3^ cells in each well), and were incubated with each treatment for appropriate time. Cell proliferation was measured using a CellTiter-blue Cell Viability Assay Kit (Promega, Madison, WI) according to the manufacturer’s instructions. Combination index (CI) was calculated [32].

### Western blot analysis

Lysate protein was extracted from whole-cells (5 × 10^6^ cells for each) after each treatment for 24 hours *in vitro* and from excised tumor tissue after treatment *in vivo*. This protocol for Western blot analysis was described in a previous study [33]. After incubating the blots in each primary antibody (1:1,000 dilution) overnight, membranes were incubated with peroxidase-labeled secondary antibody (1:10,000 dilution, Histofine; Nichirei, Tokyo, Japan) for 2 hours and detected by using Clarity Max Western ECL Substrate (BIO-RAD, Hercules, CA, USA). Protein bands were detected using a Chemi Doc XRS+ system and Image Lab Softwear (BIO-RAD).

### Cell cycle analysis

After treatment with each regimen for 48 hours, 1 × 10^5^ cells were harvested and fixed in 70% ethanol and stored at −20° C for at least 24 hours. After centrifugation, the cells were washed with PBS, and re-suspended in PBS with RNase for 4 min at 4° C. DNA was stained with propidium iodide solution (Sigma-Aldrich, St. Louis, MO, USA) for 30 minutes in the dark at 4° C. DNA content was determined with a MACSQuant R Analyzer (Miltenyi Biotec K.K., Tokyo, Japan). The data were analyzed with MACSQuantify™ Softwear version.2.5.

### Quantitative analysis of NF-κB activity

For assessment of NF-κB activity in each treatment group, the concentration of NF-κB p65 in the nuclear extracts was measured *in vitro* and *in vivo*. Nuclear extracts of treated pancreatic cells were prepared using a nuclear extract kit (Active Motif, Carlsbad, CA, USA) according to the manufacturer’s protocol. The nuclear extracts were assessed using an enzyme-linked immunosorbent assay (ELISA) kit (TransAM^TM^ NF-κB; Active Motif) to detect and quantify the NF-κB activity according to the manufacturer’s instruction.

### Immunohistochemical staining

Paraffin sections of tumor tissue were stained immunohistochemically using Ki-67 antibody (1:300) and visualized using BenchMark-XT (VENTANA Medical System, Tuson, AZ, USA). TUNEL assay was performed to evaluate the induction of apoptosis with *In Situ* Cell Death Detection Kit (Roche Diagnostics, Basel, Swiss). The percentage of Ki-67 positive cells was microscopically examined in 3 random high-power fields at 400× from three tumors. The TUNEL-positive cells were counted at 200×.

### Statistical analysis

Data were expressed as a mean ± SD. Non-paired 2-tailed *t*-test was used for statistical analysis. All *P* value were considered statistically significant at < 0.05.

## Abbreviations

Gem: gemcitabine
NF-κB: nuclear factor kappa B
IκBα: inhibitor of kappa B
IKK: IκB kinase
IAP: inhibitor of apoptosis
IMiDs: immunomodulatory drugs
TNF: tumor necrosis factor
PBS: phosphate buffer saline
ELISA: enzyme-linked immunosorbent assay.
